# Supplementary material for: Association of smoking with abdominal adipose deposition and muscle composition in Coronary Artery Risk Development in Young Adults (CARDIA) participants at mid-life: A population-based cohort study
Source: PLoS Med. 2020 Jul 21;17(7):e1003223. doi: 10.1371/journal.pmed.1003223 (PMC7373261; doi:10.1371/journal.pmed.1003223)
Supplement: S1 Table — Y25, year 25. (DOCX) [file pmed.1003223.s002.docx]

| Supplemental Table 1. Multivariable models of Y25 muscle composition [least squares mean(95% confidence interval)] by baseline smoking status | | | | | | | | |
| --- | --- | --- | --- | --- | --- | --- | --- | --- |
| Abdominal  Depot | | Model | Smoking Status | | | P _former vs never_ | P _current vs former_ | P _current vs never_ |
|  |  |  | Never | Former | Current |  |  |  |
| VAT/SAT Ratio | | Baseline | 0.448(0.435,0.460) | 0.450(0.424,0.476) | 0.481(0.462,0.500) | 0.88 | 0.059 | 0.005 |
|  |  | Y25 | 0.448(0.436,0.459) | 0.448(0.423,0.473) | 0.479(0.461,0.497) | 0.99 | 0.046 | 0.005 |
| Muscle Composition | Total Volume | Baseline | 20.4(20.3,20.6) | 20.6(20.2,20.9) | 20.5(20.3,20.8) | 0.44 | 0.91 | 0.43 |
|  |  | Y25 | 20.3(20.2,20.5) | 20.5(20.3,20.8) | 20.6(20.4,20.8) | 0.21 | 0.58 | 0.020 |
|  |  | Y25+VAT/SAT | 20.4(20.2,20.5) | 20.5(20.3,20.8) | 20.6(20.4,20.8) | 0.21 | 0.64 | 0.026 |
|  | Lean Volume | Baseline | 18.0(17.9,18.2) | 18.1(17.8,18.2) | 17.9(17.7,18.1) | 0.71 | 0.25 | 0.27 |
|  |  | Y25 | 18.0(17.9,18.1) | 18.1(17.8,18.3) | 18.0(17.8,18.1) | 0.67 | 0.55 | 0.78 |
|  |  | Y25+VAT/SAT | 18.0(17.9,18.1) | 18.1(17.8,18.3) | 18.0(17.8,18.1) | 0.67 | 0.55 | 0.67 |
|  | IMAT Volume | Baseline | 2.28(2.21,2.34) | 2.35(2.21,2.49) | 2.51(2.40,2.61) | 0.36 | 0.07 | <0.001 |
|  |  | Y25 | 2.26(2.20,2.31) | 2.36(2.25,2.47) | 2.53(2.45,2.61) | 0.09 | 0.014 | <0.001 |
|  |  | Y25+VAT/SAT | 2.26(2.21,2.31) | 2.36(2.25,2.47) | 2.53(2.44,2.61) | 0.09 | 0.021 | <0.001 |
|  | IMAT/Lean Ratio | Baseline | 0.131(0.127,0.135) | 0.133(0.125,0.141) | 0.146(0.140,0.152) | 0.73 | 0.015 | <0.001 |
|  |  | Y25 | 0.130(0.127,0.134) | 0.134(0.127,0.141) | 0.148(0.142,0.153) | 0.34 | 0.002 | <0.001 |
|  |  | Y25+VAT/SAT | 0.130(0.126,0.133) | 0.134(0.127,0.140) | 0.147(0.143,0.154) | 0.32 | 0.003 | <0.001 |
|  | Attenuation | Baseline | 41.5(41.2,41.7) | 40.9(40.3,41.5) | 40.5(40.1,40.9) | 0.07 | 0.28 | <0.001 |
|  |  | Y25 | 41.5(41.2,41.7) | 40.9(40.3,41.5) | 40.5(40.1,40.9) | 0.06 | 0.22 | <0.0001 |
|  |  | Y25+VAT/SAT | 41.5(41.2,41.7) | 40.9(40.4,41.4) | 40.5(40.2,40.9) | 0.06 | 0.29 | <0.001 |

Baseline model (baseline smoking and covariate adjustment): age, race, sex, center, education, physical activity, alcohol consumption, fast food consumption, systolic BP, triglycerides, glucose, and BMI; Y25 model (Y25 smoking status and covariate adjustment): age, race, sex, center, education, physical activity, alcohol consumption, sugar-sweetened soda consumption, fast food consumption, diabetes, cholesterol treatment, hypertension treatment, systolic BP, triglycerides, C-reactive protein, prevalent coronary artery calcification, and BMI (+ VAT/SAT Ratio for muscle composition variables); tissue volumes are in cm^3^ and attenuation is in Hounsfield Units (HU).
