# Supplementary material for: Association of smoking with abdominal adipose deposition and muscle composition in Coronary Artery Risk Development in Young Adults (CARDIA) participants at mid-life: A population-based cohort study
Source: PLoS Med. 2020 Jul 21;17(7):e1003223. doi: 10.1371/journal.pmed.1003223 (PMC7373261; doi:10.1371/journal.pmed.1003223)
Supplement: S2 Table — Y25, year 25. (DOCX) [file pmed.1003223.s003.docx]

| Supplemental Table 2. Multivariable models of Y25 muscle composition [least square mean(95% confidence interval)] by baseline cotinine level | | | | | |
| --- | --- | --- | --- | --- | --- |
| Measure | | Model | Cotinine (ng/ml) | | P |
|  |  |  | <14 ng/ml | ≥14 ng/ml |  |
| VAT/SAT Ratio | | Baseline | 0.449(0.438,0.461) | 0.473(0.454,0.492) | 0.037 |
|  |  | Y25 | 0.449(0.439,0.460) | 0.471(0.453,0.489) | 0.045 |
| Muscle Composition | Total Volume | Baseline | 20.5(20.3,20.6) | 20.5(20.2,20.7) | 0.98 |
|  |  | Y25 | 20.4(20.3,20.5) | 20.5(20.4,20.7) | 0.20 |
|  |  | Y25 + VAT/SAT | 20.4(20.3,20.5) | 20.5(20.4,20.7) | 0.24 |
|  | Lean Volume | Baseline | 18.1(17.9,18.2) | 17.9(17.7,18.1) | 0.12 |
|  |  | Y25 | 18.0(17.9,18.1) | 17.9(17.8,18.1) | 0.37 |
|  |  | Y25 + VAT/SAT | 18.0(17.9,18.1) | 17.9(17.8,18.1) | 0.37 |
|  | IMAT Volume | Baseline | 2.30(2.24,2.36) | 2.47(2.37,2.57) | 0.004 |
|  |  | Y25 | 2.28(2.24,2.33) | 2.50(2.42,2.58) | <0.001 |
|  |  | Y25 + VAT/SAT | 2.29(2.24,2.33) | 2.50(2.42,2.57) | <0.001 |
|  | IMAT/Lean Ratio | Baseline | 0.132(0.128,0.136) | 0.144(0.138,0.150) | 0.001 |
|  |  | Y25 | 0.131(0.128,0.134) | 0.146(0.141,0.151) | <0.001 |
|  |  | Y25 + VAT/SAT | 0.131(0.128,0.135) | 0.146(0.141,0.151) | <0.001 |
|  | Attenuation | Baseline | 41.4(41.1,41.6) | 40.5(40.1,40.9) | <0.001 |
|  |  | Y25 | 41.4(41.2,41.6) | 40.5(40.1,40.9) | <0.001 |
|  |  | Y25 + VAT/SAT | 41.4(41.2,41.6) | 40.5(40.2,40.9) | <0.001 |

Baseline model (baseline cotinine and covariate adjustment): age, race, sex, center, education,

physical activity, alcohol consumption, fast food consumption, systolic BP, triglycerides, glucose,

and BMI; Y25 model (baseline cotinine and Y25 covariate adjustment): age, race, sex, center, education, physical activity, alcohol consumption, sugar-sweetened soda consumption, fast food consumption, diabetes, cholesterol treatment, hypertension treatment, systolic BP, triglycerides, C-reactive protein, prevalent coronary artery calcification , and BMI (+ VAT for muscle composition variables); tissue volumes are in cm^3^ and attenuation is in Hounsfield Units (HU).
