# Supplementary material for: Association of smoking with abdominal adipose deposition and muscle composition in Coronary Artery Risk Development in Young Adults (CARDIA) participants at mid-life: A population-based cohort study
Source: PLoS Med. 2020 Jul 21;17(7):e1003223. doi: 10.1371/journal.pmed.1003223 (PMC7373261; doi:10.1371/journal.pmed.1003223)
Supplement: S1 CARDIA Proposal — CARDIA, Coronary Artery Risk Development in Young Adults. (DOCX) [file pmed.1003223.s004.docx]

**Manuscript Proposal, CARDIA Study**

| **Date Submitted: 02/22/2018** |
| --- |

**(Page 2 - Instructions to complete and submit proposal. Delete instructions page prior to submitting proposal**)

| **I. Title:** | Association of Smoking with Abdominal Muscle Composition in Mid-Life: The CARDIA Study |
| --- | --- |
|  |  |

| **II. Abbreviated Title:** | Smoking and Muscle Composition |
| --- | --- |

**Select the type of manuscript:** **type an “X” in the box to the “RIGHT” of the type** (these are not check-boxes)

| **III.** | **Data: Core**: | **X** | **Ancillary**: | **X** | **Substudy**: |  | **Genetic:** |  |  |
| --- | --- | --- | --- | --- | --- | --- | --- | --- | --- |
|  | ***Include study name below, as applicable:*** | | | | | | | | |
|  | Ancillary Study: Longitudinal changes in pericardial adiposity and subclinical atherosclerosis: The CARDIA study | | | | | | | | |
|  | Substudy: | | | | | | | | |
|  | Keywords: smoking, muscle composition, CT, adipose tissue | | | | | | | | |

***Reminder: Identify your keywords!***

**Select data years to be used in proposal: type an “X” in the box to the “RIGHT” of exam** (these are not check-boxes)

| **IV.** | **Year 0** | | **Year 2** | | **Year 5** | | **Year 7** | | **Year 10** | | **Year 15** | | **Year 20** | | **Year 25** | | **Year 30** | |
| --- | --- | --- | --- | --- | --- | --- | --- | --- | --- | --- | --- | --- | --- | --- | --- | --- | --- | --- |
| **Data:** | **Exam 1** | **X** | **Exam 2** | **X** | **Exam 3** | **X** | **Exam 4** | **X** | **Exam 5** | **X** | **Exam 6** | **X** | **Exam 7** | **X** | **Exam 8** | X | **Exam 9** |  |
|  | **1985-86** | | **1987-88** | | **1990-91** | | **1992-93** | | **1995-96** | | **2000-01** | | **2005-06** | | **2010-11** | | **2015-16** | |

**V. Writing Group**

- **Core and Ancillary Study manuscript proposals:** Include **ALL** authors in the area below; **DO NOT** type their names here.
- **Genetics manuscript proposals only:** Include in authors area below: (1) first 3 authors, (2) CARDIA Representative, (3) all CARDIA authors, and (4) last author
- **As of Apr 6, 2016: “Proposals submitted to P&P should have at most five (5) co-authors from one center (and preferably fewer), and no more than nine (9) total authors, to allow for collaborators from other centers to join**.”

| **First author**: | Greg Terry | | | E-mail: [james.g.terry@vanderbilt.edu](mailto:james.g.terry@vanderbilt.edu) | Phone: | 615.322.6462  615.875.8986 |
| --- | --- | --- | --- | --- | --- | --- |
| Affiliation/Address: | | Radiology and Radiological Sciences, Vanderbilt University Medical Center (VUMC), Nashville, TN, 2525 West End Ave, Suite 300-B, Nashville, TN 37203 | | | | |
|  | | | | | | |
| First author CARDIA Center Affiliation: | | | **Vanderbilt CT Reading Center** | | | |

Example: Coordinating Center, Birmingham Field Center, Echo Reading Center, etc.

| ***CARDIA Representative**: | Jeff Carr | E-mail: | [j.jeffrey.carr@vanderbilt.edu](mailto:j.jeffrey.carr@vanderbilt.edu) |
| --- | --- | --- | --- |

*A Representative is a CARDIA Investigator who is Steering Committee approved to represent CARDIA in publications and who works closely with you on this manuscript. See instructions for link to “Representatives” list.

**Co-authors**: Name, affiliation, city, state, country, and e-mail (**type left to right**). Katherine G. Hartley, Radiology and Radiological Sciences, Vanderbilt University Medical Center, Nashville, TN, [kate.hartley@vanderbilt.edu](mailto:kate.hartley@vanderbilt.edu); Sangeeta Nair, Radiology and Radiological Sciences, Vanderbilt University Medical Center, Nashville, TN, [sangeeta.nair@vanderbilt.edu](mailto:sangeeta.nair@vanderbilt.edu); Lyn M. Steffen, Division of Epidemiology & Community Health, University of Minnesota, Minneapolis, MN, [steff025@umn.edu](mailto:steff025@umn.edu); Melissa F. Wellons, Division of Diabetes, Endocrinology, and Metabolism, Vanderbilt University Medical Center, Nashville, TN, [melissa.wellons@vanderbilt.edu](mailto:melissa.wellons@vanderbilt.edu); David R. Jacobs, Jr., Division of Epidemiology & Community Health, University of Minnesota, Minneapolis, MN, [jacob004@umn.edu](mailto:jacob004@umn.edu); J. Jeffrey Carr, Radiology and Radiological Sciences, Vanderbilt University Medical Center, Nashville, TN, [j.jeffrey.carr@vanderbilt.edu](mailto:j.jeffrey.carr@vanderbilt.edu); Hilary A. Tindle, Department of Medicine, Vanderbilt University Medical Center, Nashville, TN, [hilary.tindle@vanderbilt.edu](mailto:hilary.tindle@vanderbilt.edu)

**VI. Background**

Age-related loss of skeletal muscle may lead to clinically-significant sarcopenia, placing older adults at higher risk of falls and fractures (Marty et al. 2017). Declines in total muscle mass associated with aging are more specifically a reduction in lean muscle tissue with an accompanying relative increase intermuscular adipose tissue (IMAT), a minor component of healthy muscles (Marty et al. 2017; Miljkovic and Zmuda 2010). Incremental loss of lean muscle and attendant increases in IMAT with aging may also contribute to development of insulin resistance, hypertension and hyperlipidemia, all of which increase cardiovascular disease (CVD) risk (Miljkovic and Zmuda 2010; Therkelsen et al. 2013). We recently showed in CARDIA that higher IMAT was associated with prevalent coronary artery calcium (CAC) in midlife (Terry et al. 2017) and other researchers have shown that IMAT accumulation increases risk of CVD and total mortality in the elderly (Miljkovic et al. 2015).

Though clinically-significant muscle loss would be expected to be rare in CARDIA participants at Y25 (43-55 years of age), these participants are squarely within the age range during which subclinical loss of muscle mass accelerates (Marty et al. 2017). It is therefore important to identify modifiable risk factors contributing to muscle loss and detrimental changes in muscle composition at these earlier stages. Although higher BMI is associated with larger muscle mass, studies have shown that obesity contributes to higher muscle-associated fat (Curtis et al. 2015). Generalized obesity and central adipose deposition are strongly associated with sedentary lifestyles (Larsen et al. 2014) which are in turn linked to loss of skeletal muscle mass (Curtis et al. 2015; Marty et al. 2017).

Although smoking is associated with lower physical activity and reduced exercise capacity (Sidney et al. 1993), its association with obesity as measured using BMI is paradoxical. A recent cross-sectional study in almost half a million participants reported that current smokers were 17% less likely to be obese than never smokers; and, moreover, former smokers were 33% more likely to be obese than current smokers (Dare, Mackay, and Pell 2015). Women’s Health Initiative (WHI) participants who were smokers at enrollment, but quit during the first 3 years of follow-up gained an average of 4.4 kg more than women who continued to smoke over the 6-year course of the clinical trial (Kabat et al. 2017). Although these studies explain the rationale for not quitting among some smokers, there are potentially deleterious changes in adipose tissue distribution and specific adipose depots that accompany smoking. After accounting for BMI, waist and waist-hip ratio were higher, indicating a more centralized adipose distribution, in current and former smokers compared to never smokers in a cross-sectional analysis from European Prospective Investigation in Cancer (EPIC) (Canoy et al. 2005). Deposition of excess fat centrally as visceral adipose tissue (VAT) or within or about non-adipose tissues or organs (e.g. liver, muscle, or heart) is strongly associated with diabetes and CVD risk (Després 2012). Smoking is associated with VAT in some, but not all, studies to date, although very little is known about the role of smoking in adipose-related changes in muscle or other non-adipose tissues (Hairston et al. 2012; Kim et al. 2012; Nakanishi et al. 2014). There is some evidence that smoking is associated with lower muscle mass and/or poor muscle function from relatively small studies that were not designed to answer questions about muscle composition changes (van den Borst et al. 2011; Kok, Hoekstra, and Twisk 2012).

Using the CT measures of abdominal muscles and adipose depots available at Y25, we will examine the role of current smoking status and smoking history in abdominal muscle composition (e.g. IMAT volume, lean and IMAT as proportions of total muscle volume, and total muscle volume). We will further examine so-called muscle quality using attenuation values for total, lean and adipose components of abdominal muscles. Our overarching hypothesis is that muscle lean tissue volume will be lower and IMAT higher in current and former smokers compared to never smokers after adjusting for generalized obesity and other risk factors associated with muscle composition.

**VII. Main Study Questions and Hypotheses**

**Hypothesis 1:** We hypothesize that absolute IMAT volume and proportion of IMAT will be higher in current smokers than never smokers (with former smokers intermediate) after adjustment for age, race, sex, physical activity and other potential contributors to muscle composition including BMI or VAT.

**Hypothesis 2:** We hypothesize that CT attenuation values (Hounsfield Units) will be lower (indicating lower muscle quality) in abdominal muscles in smokers versus never smokers (with former smokers intermediate)after adjustment for age, race, sex, physical activity and other potential contributors to muscle composition including BMI or VAT.

**VIII. CARDIA data to be used (Required: Exam year(s):** Y20, Y25; **etc.) - See page 2 for instructions.**

We require smoking status/history and BMI at each exam year. We also require cotinine levels at obtained only at Y0.

At Y25, we require CT abdominal fat and muscle variables and model adjustment variables including age, race, sex, center,

education, alcohol consumption, dietary variables including fast food and sugar-sweetened beverage consumption,

physical activity, cholesterol and hypertension treatment status, blood pressures, glucose, 2hr-glucose, HbA1c,diabetes treatment status, fasting status, HDL-cholesterol, triglycerides, CRP, and BMI. All variables are available at the CT Reading Center.

**IX. Analytic Plan**

Our primary outcome variable will be continuous IMAT volume, but we will also examine the continuous dependent variables of IMAT/total muscle volume ratio and muscle total attenuation, lean attenuation, and fat attenuation in subsequent models. Overall muscle composition (mean values of left and right side psoas, paraspinous, lateral oblique, and rectus muscles) will be the primary outcome, but we will also examine paraspinous and psoas muscles separately as these muscles are more commonly measured in studies to date. We will evaluate key variable distributions for adherence to assumptions for linear models and transform variables if necessary to better reflect the normal distribution. We will test associations of continuous muscle measures with smoking status (never/former/present) in unadjusted analyses and multivariable linear models adjusted for potential confounders. Along with smoking status, the primary multivariable model (model 1) will include age, race, sex, center, education, physical activity,alcohol consumption, and fast food (FF) and sugar-sweetened beverage (SSB) consumption. Model 2 will include model 1 variables plus hypertension and cholesterol treatment status; systolic blood pressure; diabetes status; triglyceride; and CRP. Models 3a and 3b will include model 2 components along with BMI and VAT, respectively. We will next repeat all models described using the independent variables total years smoking exposure derived from smoking status at each CARDIA visit. We will also examine possible associations of pack-years of smoking and years since quitting to IMAT in the subset of those who ever smoked. We will test for sex, race interactions in each model. All analyses will be performed using STATA 15.

**X. Timeline**

P&P Proposal Review: March 2018

Analyses: March 2018

Abstract Drafting and P&P Review: March-April 2018

Manuscript Drafting and Co-author Review: March-June 2018

P&P Manuscript Review: July 2018

Journal Submission: August-September 2018

**References (limit: one page)**

van den Borst, B. et al. 2011. “Is Age-Related Decline in Lean Mass and Physical Function Accelerated by Obstructive Lung Disease or Smoking?” *Thorax* 66(11): 961–69. http://thorax.bmj.com/cgi/doi/10.1136/thoraxjnl-2011-200010.

Canoy, D et al. 2005. “Cigarette Smoking and Fat Distribution in 21,828 British Men and Women: A Population-Based Study.” *Obes Res* 13(8): 1466–75. http://www.ncbi.nlm.nih.gov/pubmed/16129730%5Cnhttp://onlinelibrary.wiley.com/store/10.1038/oby.2005.177/asset/oby.2005.177.pdf?v=1&t=irjz2l17&s=55431dd59761c83c07cc4fa5d73e311f2de61ada.

Curtis, Elizabeth, Anna Litwic, Cyrus Cooper, and Elaine Dennison. 2015. “Determinants of Muscle and Bone Aging.” *Journal of Cellular Physiology* 230(11): 2618–25.

Dare, Shadrach, Daniel F Mackay, and Jill P Pell. 2015. “Relationship between Smoking and Obesity: A Cross-Sectional Study of 499,504 Middle-Aged Adults in the UK General Population.” *PLOS ONE* 10(4): 1–12. https://doi.org/10.1371/journal.pone.0123579.

Després, Jean-Pierre. 2012. “Body Fat Distribution and Risk of Cardiovascular Disease: An Update.” *Circulation* 126(10): 1301–13. http://www.ncbi.nlm.nih.gov/pubmed/22949540 (August 7, 2014).

Hairston, Kristen G. et al. 2012. “Lifestyle Factors and 5-Year Abdominal Fat Accumulation in a Minority Cohort: The IRAS Family Study.” *Obesity* 20(2): 421–27. http://dx.doi.org/10.1038/oby.2011.171/nature06264.

Kabat, Geoffrey C et al. 2017. “Smoking Habits and Body Weight Over the Adult Lifespan in Postmenopausal Women.” *American journal of preventive medicine* 52(3): e77–84.

Kim, Jung Hwan et al. 2012. “Cigarette Smoking Increases Abdominal and Visceral Obesity but Not Overall Fatness: An Observational Study.” *PLOS ONE* 7(9): 1–5. https://doi.org/10.1371/journal.pone.0045815.

Kok, Maarten O, Trynke Hoekstra, and Jos W R Twisk. 2012. “The Longitudinal Relation between Smoking and Muscle Strength in Healthy Adults.” *European addiction research* 18(2): 70–75.

Larsen, Britta A et al. 2014. “Associations of Physical Activity and Sedentary Behavior with Regional Fat Deposition.” *Medicine and science in sports and exercise* 46(3): 520–28.

Marty, Eric et al. 2017. “A Review of Sarcopenia: Enhancing Awareness of an Increasingly Prevalent Disease.” *Bone* 105: 276–86.

Miljkovic, Iva et al. 2015. “Greater Skeletal Muscle Fat Infiltration Is Associated With Higher All-Cause and Cardiovascular Mortality in Older Men.” *The Journals of Gerontology Series A: Biological Sciences and Medical Sciences*. http://biomedgerontology.oxfordjournals.org/content/early/2015/04/01/gerona.glv027.abstract.

Miljkovic, Iva, and Joseph M Zmuda. 2010. “Epidemiology of Myosteatosis.” *Current opinion in clinical nutrition and metabolic care* 13(3): 260–64.

Nakanishi, Kaori et al. 2014. “Smoking Associates with Visceral Fat Accumulation Especially in Women.” *Circulation journal : official journal of the Japanese Circulation Society* 78(5): 1259–63.

Sidney, S et al. 1993. “Cigarette Smoking and Submaximal Exercise Test Duration in a Biracial Population of Young Adults: The CARDIA Study.” *Medicine and science in sports and exercise* 25(8): 911–16.

Terry, James G et al. 2017. “Intermuscular Adipose Tissue and Subclinical Coronary Artery Calcification in Midlife: The CARDIA Study (Coronary Artery Risk Development in Young Adults).” *Arteriosclerosis, thrombosis, and vascular biology* 37(12): 2370–78.

Therkelsen, Kate E et al. 2013. “Intramuscular Fat and Associations With Metabolic Risk Factors in the Framingham Heart Study.” *Arteriosclerosis, Thrombosis, and Vascular Biology* 33(4): 863–70. http://atvb.ahajournals.org/content/33/4/863.abstract.

**Submit Manuscript Proposal to:** [lsellers@uabmc.edu](mailto:lsellers@uabmc.edu)

**Instructions to Complete and Submit Manuscript Proposal, CARDIA Study**

1. It is recommended that a manuscript proposal be limited to three (3) pages, excluding references. Limit references to one page. **Required Font**: Calibri 10 or 11 pt (Do not use bold text in proposal.)

**Document should be named as follows**: First authors last name_first initial_abbreviated title_current date, i.e., “Kiefe_C_Smoking and SE factors_12-30-2015. **Document may be returned to author if it is not named as requested**.

2. **Section V: Writing Group**:

- **Core and Ancillary Study manuscript proposals:** Include all authors - see additional instructions on proposal.
- **Genetics manuscript proposals only**: include authors as specified on proposal.
- **As of Apr 6, 2016: “Proposals submitted to P&P should have at most five (5) co-authors from one center (and preferably fewer), and no more than nine (9) total authors, to allow for collaborators from other centers to join**.”
- **Provide contact information for the first author and co-authors**.
- **NOTE**: A “Representative” is a CARDIA Investigator working closely with you on this manuscript. CARDIA “Representatives” list is posted: <http://www.cardia.dopm.uab.edu/publications-2/publications-documents>s
- **First Author**: Include complete contact information, i.e., Affiliation/address, e-mail and phone.
- **Co-Authors**: Name, affiliation, city, state, country, and e-mail; type left to right on manuscript proposal form

3. **Section VIII. CARDIA data to be used (i.e., Exam year(s):** Y20, Y25**; etc.)**

You selected the data years in **Section IV** on page 1 of proposal. You may relist these in this section and provide a summary of the data type which you will use (summary limited to 10-12 sentences).

**NOTE:** Dataset Request - Forms/Variables: *These are not included on manuscript proposal, but are included in an analytic dataset request (DSR) submitted to the Coordinating Center.* See page 1 of the DSR: instructions to complete/submit the data request: <http://www.cardia.dopm.uab.edu/publications-2/publications-documents>.

4. **Review/approval of proposal prior to submission to Publications and Presentations (P&P) Subcommittee*:***

The CARDIA “Representative” and all authors must review/approve the manuscript proposal prior to submission to the *CARDIA P&P Subcommittee.*

5. **Emailing proposal to Coordinating Center (CC)**: Email to [lsellers@uabmc.edu](mailto:lsellers@uabmc.edu) with a request for review on a P&P call.

- Include in the email message: Title, authorship, CARDIA Representative, selected call date, and “email addresses” for authors who will receive comments.
- A manuscript proposal is due to the Coordinating Center the Wednesday prior to the Wednesday on which the proposal will be reviewed. The first author or a co-author must attend the call on which the proposal is reviewed.

6. **P&P Calls are the 2^nd^. & 4^th^. Wednesday of each month, 2:00 – 3:00 pm (CT). To connect:** (1) Dial 1-866-244-8528; (2) Passcode: 901921; 3) Once connected, announce yourself. Drs. Catarina Kiefe and Donald Lloyd-Jones, Chairs

7. The Publications Policy, and all other publications associated documents and forms are available on the CARDIA Public Website: <http://www.cardia.dopm.uab.edu/publications-2/publications-documents>.

All documents and forms are PDF or Word. The Word files may be downloaded and completed. The DMDA (PDF) has fields in which to enter the information. All forms are emailed to [lsellers@uabmc.edu](mailto:lsellers@uabmc.edu) for processing.

8. Questions? Email [lsellers@uabmc.edu](mailto:lsellers@uabmc.edu)

**All of the requested information is necessary; the proposal may be returned if incomplete**.

***Thank you for your interest in the CARDIA Study!***
