# Supplementary material for: Association of smoking with abdominal adipose deposition and muscle composition in Coronary Artery Risk Development in Young Adults (CARDIA) participants at mid-life: A population-based cohort study
Source: PLoS Med. 2020 Jul 21;17(7):e1003223. doi: 10.1371/journal.pmed.1003223 (PMC7373261; doi:10.1371/journal.pmed.1003223)
Supplement: S1 Form — CARDIA, Coronary Artery Risk Development in Young Adults. (PDF) [file pmed.1003223.s005.pdf]

TOBACCO USE QUESTIONNAIRE
CARDIA VIII—Year 25 Exam

H10EXDATE *H10SDATE*

Exam Date: ___ ___/___ ___/___ ___ ___ ___ **OR** Same Date Blood Pressure Taken ❑

1. Have you ever used any tobacco product such as cigarettes, cigars, tobacco pipe, chewing tobacco, snuff, nicotine chewing gum, or a nicotine patch? ***H10TOBAC***

   1 ☐ No → GO TO QUESTION 8
   2 ☐ Yes

2. Have you ever smoked cigarettes regularly for at least three months? By "regularly" we mean at least 5 cigarettes per week almost every week. ***H10CIGS***

   1 ☐ No → COMPLETE FORM 9-TOB, QUESTION 2.02
   2 ☐ Yes → COMPLETE FORM 9-TOB, QUESTION 2.01

3. Have you ever smoked cigars regularly for at least three months? By "regularly" we mean at least 2 cigars per week almost every week. ***H10CIGAR***

   1 ☐ No
   2 ☐ Yes → COMPLETE FORM 9-TOB, QUESTION 3.01

4. Have you ever smoked a tobacco pipe regularly for at least three months? By "regularly" we mean at least 2 pipes full of tobacco a week almost every week. ***H10PIPE***

   1 ☐ No
   2 ☐ Yes → COMPLETE FORM 9-TOB, QUESTION 4.01

5. Have you regularly used smokeless tobacco such as snuff, chewing tobacco or other product such as *Skoal Bandits* for at least three months? By "regularly" we mean at least once a week almost every week. ***H10SMKLS***

   1 ☐ No
   2 ☐ Yes → COMPLETE FORM 9-TOB, QUESTION 5.01

6. Have you ever used a nicotine gum such as *Nicorette*? ***H10GUMEV***

   1 ☐ No
   2 ☐ Yes → 6a. Have you used it in the past week?

      1 ☐ No ***H10GUMWK***
      2 ☐ Yes

___ ___ ___ INTERVIEWER ID ***H10IVID***

7. Have you ever used a nicotine patch such as *Nicoderm* or *Habitrol*? ***H10PATEV***

1☐ No
2☐ Yes → 7a. Have you used it in the past week? ***H10PATWK***

   1☐ No
   2☐ Yes

8. Does your spouse or significant other currently smoke cigarettes? ***H10SPSMK***

1☐ No
2☐ Yes
9☐ Not applicable (YOU DO NOT HAVE A SPOUSE/SIGNIFICANT OTHER)

9. Not counting yourself, how many people do you live with who smoke cigarettes? ***H10LVHME***

☐☐ (RECORD "00" IF YOU LIVE ALONE)

10. On the average, how many hours per week are you exposed to cigarette, cigar, or pipe smoke in your home because of smoking by others? (ROUND DOWN) ***H10EXHME***

☐☐ hours per week

11. On the average, how many hours per week are you exposed to cigarette, cigar, or pipe smoke in a small space other than your home (such as airplane, office, car, etc.) because of smoking by others? (ROUND DOWN) ***H10EXSML***

☐☐ hours per week

12. On the average, how many hours per week are you exposed to cigarette, cigar, or pipe smoke in a large indoor area (such as restaurant, hotel lobby, lecture hall, etc.) because of smoking by others? (ROUND DOWN) ***H10EXLRG***

☐☐ hours per week

13. Is there a smoking policy which limits smoking or does not allow smoking at your workplace? ***H10SMKPL***

1☐ No policy
2☐ There is a policy which <u>limits</u> smoking at work
3☐ There is a policy which <u>prohibits</u> smoking where I work
8☐ Don't know
9☐ Not applicable, don't work (full-time or part-time) in an office environment around others indoors
