# Supplementary material for: Association of smoking with abdominal adipose deposition and muscle composition in Coronary Artery Risk Development in Young Adults (CARDIA) participants at mid-life: A population-based cohort study
Source: PLoS Med. 2020 Jul 21;17(7):e1003223. doi: 10.1371/journal.pmed.1003223 (PMC7373261; doi:10.1371/journal.pmed.1003223)
Supplement: S2 Form — CARDIA, Coronary Artery Risk Development in Young Adults. (PDF) [file pmed.1003223.s006.pdf]

9-TOB

FOLLOW-UP QUESTIONS FOR TOBACCO QUESTIONNAIRE
CARDIA VIII—Year 25 Exam

H09KEXDT
Exam Date: ___ ___/___ ___/___ ___ ___ ___ **OR** *H09KSDATE* Same Date Blood Pressure Taken ❑

IF THE PARTICIPANT ANSWERED *YES* OR *NO* TO QUESTION 2, *YES* TO QUESTION 3, *YES* TO QUESTION 4, OR *YES* TO QUESTION 5 ON FORM 10, COMPLETE THIS FORM.

FORM 10 QUESTION 2 FOLLOW-UP QUESTIONS

► **Form 10 Question 2 *YES* response follow-up**

2.01 Do you still smoke cigarettes regularly? By "regularly" we mean at least 5 cigarettes per week, almost every week. *H09SMKNW*

1 ☐ No →
2.01a. How long has it been since you smoked cigarettes regularly?
H09QTNUM *H09QTFRQ*
☐☐ 1 ☐ Days 2 ☐ Weeks 3 ☐ Months 4 ☐ Years

2.01b. How old were you when you started smoking cigarettes regularly?
☐☐ years *H09SMKAG1*

2.01c. Altogether, how many years did you smoke cigarettes regularly?
☐☐ years *H09SMKNCR1*

If *YES* TO Q3, Q4, OR Q5 ON FORM 10, GO TO PAGE 4, OTHERWISE END OF QUESTIONNAIRE

2 ☐ Yes → GO TO QUESTION 2.03

► **Form 10 Question 2 *NO* response follow-up**

2.02 Have you started smoking regularly in the last three months? By "regularly" we mean at least 5 cigarettes per week, almost every week. *H09STS3M*

1 ☐ No → If *YES* TO Q3, Q4, OR Q5 ON FORM 10, GO TO PAGE 4, OTHERWISE END OF QUESTIONNAIRE

2 ☐ Yes →
2.02a. Do you still smoke cigarettes regularly now? *H09SLS3M*
(NOW = WITHIN THE PAST WEEK)
1 ☐ No → If *YES* TO Q3, Q4, OR Q5 ON FORM 10, GO TO PAGE 4, OTHERWISE END OF QUESTIONNAIRE
2 ☐ Yes

___ ___ ___ INTERVIEWER ID *H09KIVID*

2.03 How many cigarettes do you smoke per day on the average? ***H09CGTDY***

☐☐ cigarettes per day (1 pack = 20 cigarettes)

2.04 Do you now smoke cigarettes every day or just some days? ***H09SMKDY***

1 ☐ Every day
2 ☐ Some days, not every day →

> 2.04a. On how many of the past 30 days did you smoke cigarettes?
>
> ☐☐ days ***H09SMK30***
>
> 2.04b. On the days that you smoke, about how many cigarettes do you usually smoke per day?
>
> ☐☐ cigarettes per day ***H09CIGDY***

2.05 How old were you when you started smoking cigarettes regularly? ***H09SMKAG***

☐☐ years

2.06 Altogether, how many years have you smoked cigarettes regularly? ***H09SMKNCR***

☐☐ years

2.07 For your current brand of cigarettes:

2.07a. Are they filtered or unfiltered? ***H09FILTR***

1 ☐ Filtered
2 ☐ Unfiltered
8 ☐ Don't know

2.07b. Are they regular, king size, long, or extra long? ***H09LNGTH***

1 ☐ Regular (70 mm)
2 ☐ King (85 mm)
3 ☐ Long (100 mm)
4 ☐ Extra long (120 or 125 mm)
8 ☐ Don't know

2.07c. Are they menthol or non-menthol? ***H09MENTH***

1 ☐ Menthol
2 ☐ Non-menthol
8 ☐ Don't know

2.08 Have you made any attempts to stop smoking cigarettes in the past five years? ***H09TRYQT***

1 ☐ No
2 ☐ Yes →

> 2.08a. How many of these attempts lasted 48 hours or more? ***H09ATTMP***
>
> 1 ☐ None
> 2 ☐ 1
> 3 ☐ 2 - 3
> 4 ☐ 4 - 5
> 5 ☐ 6 or more

2.09 Do you plan to make any of these changes in your smoking in the next six months?

No	Yes	
1 ☐	2 ☐	Quit completely ***H09CHGQT***
1 ☐	2 ☐	Cut down on number of cigarettes smoked ***H09CHGNO***
1 ☐	2 ☐	Change to lower "tar" or nicotine cigarette ***H09CHGLO***
1 ☐	2 ☐	Any other changes that we did not mention? ***H09CHGOT***

> 2.09a. What are these changes? ***H09CHNGS*** ____________________

2.10 On a scale of 0 to 10, where 0 means not at all interested in quitting and 10 means very interested, how interested are you in quitting smoking? ***H09QUIT***

☐☐

If *YES* TO Q3, Q4, OR Q5 ON FORM 10, GO TO PAGE 4, OTHERWISE END OF QUESTIONNAIRE

9-TOB

FOLLOW-UP QUESTIONS FOR TOBACCO QUESTIONNAIRE
CARDIA VIII—Year 25 Exam

FORM 10 <u>QUESTION 3</u> *YES* RESPONSE FOLLOW-UP QUESTION

3.01 Do you still smoke cigars regularly? ***H09CIGAR***

1 ☐ No
2 ☐ Yes
3 ☐ No, but I still smoke cigars occasionally

FORM 10 <u>QUESTION 4</u> *YES* RESPONSE FOLLOW-UP QUESTION

4.01 Do you still smoke a pipe regularly? ***H09PIPE***

1 ☐ No
2 ☐ Yes
3 ☐ No, but I still smoke a pipe occasionally

FORM 10 <u>QUESTION 5</u> *YES* RESPONSE FOLLOW-UP QUESTION

5.01 Do you still use smokeless tobacco? ***H09SNUFF***

1 ☐ No
2 ☐ Yes
