# Supplementary material for: Association of smoking with abdominal adipose deposition and muscle composition in Coronary Artery Risk Development in Young Adults (CARDIA) participants at mid-life: A population-based cohort study
Source: PLoS Med. 2020 Jul 21;17(7):e1003223. doi: 10.1371/journal.pmed.1003223 (PMC7373261; doi:10.1371/journal.pmed.1003223)
Supplement: Attachment — Submitted filename: PLOS Med Editor and Reviewer Response.docx [file pmed.1003223.s007.docx]

**Requests from the editors:**

- **Please ensure that the paper adheres to the PLOS Data Availability Policy Response:** We now include the following statement regarding data availability beginning at line 493 of the revision markup.

Data Availability

Data are available from the CARDIA Coordinating Center: <http://www.cardia.dopm.uab.edu/contact-cardia>. A description of the NHLBI policies governing the data and describing access to the data can be found at the following website: <http://www.cardia.dopm.uab.edu/study-information/nhlbi-data-repository-data>. Others have access to these data in the same manner as the authors and the authors do not have any special access privileges that others would not have.

- **Please revise title according to *PLOS Medicine’s* style.** Response: As requested, we have revised the manuscript title to include the phrase “prospective cohort” and the title already adheres to the other aspects of the PLOS Medicine style. The revised title is: “Smoking is associated with abdominal adipose deposition and muscle composition in CARDIA Study participants at mid-life: a prospective cohort study”.
- **In the last sentence of the Abstract Methods and Findings section, please describe the main limitation(s) of the study's methodology - in the main discussion section the possibility of residual confounding is mentioned, but reverse causality (as noted by one reviewer) might also be a potential source of bias, and this could be discussed/mentioned as appropriate in either the main Discussion section or abstract, as appropriate.** Response: As requested, we have modified the Abstract to include the sentence “These findings must be viewed with caution as residual confounding and/or reverse causation may contribute to these associations” at the end of the Methods and Findings section (line 51 of revision markup). We believe these are the main limitations of the study. Also, as requested by Reviewer #2, we have specifically addressed the possibility of reverse causality in the Limitations section (Line 465 of revision). This section already indirectly addressed this possibility in stating that we assessed smoking exposure multiple times prior to year 25, but the abdominal measures only once at visit year 25 (see also response to Reviewer #2 below).
- **At this stage, we ask that you include a short, non-technical Author Summary of your research to make findings accessible to a wide audience that includes both scientists and non-scientists. The Author Summary should immediately follow the Abstract in your revised manuscript.** Response: As requested, we include a short, non-technical Author Summary in the revised manuscript (see lines 63-89). We hope this summary will meet the ***PLOS Medicine*** requirements but welcome any suggestions for changes.
- **We'd ask you to modify the referencing format to match *PLOS Medicine's* style (this should be numbered callouts in square brackets (eg [1, 2]) rather than superscript numerals.** Response: As requested, we have changed the reference format to meet ***PLOS Medicine’s*** style***.***
- **Did your study have a prospective protocol or analysis plan? Please state this (either way) early in the Methods section.** Response: The analysis plan for the present manuscript was drafted as part of the manuscript proposal required of all CARDIA proposals. We now include details of the CARDIA proposal requirements within the revised manuscript beginning at line 146. Moreover, we included details of the analyses in the Statistics section of the revised manuscript beginning on line 245 and these correspond exactly with both the CARDIA proposal and the manuscript.
- **In the Abstract, currently effect estimates for differences between compared groups are presented, along with p-values. The journal normally expects that 95% confidence intervals (CI's) will also be given so that readers can understand the imprecision associated with the effects seen in the study. We'd ask that these are given both in the abstract and in the main text along with the main effect estimates.** **Response:** As requested, the model estimate is now presented with 95% confidence intervals in the Abstract and Table 3, Figure 1 legend, and Supplemental Tables 1 and 2.
- **As the paper reports findings from a prospective cohort, we'd suggest that the study is reported according to the STROBE guideline; please include the completed STROBE checklist as Supporting Information. Please add the following statement, or similar, to the Methods: "This study is reported as per the Strengthening the Reporting of Observational Studies in Epidemiology (STROBE) guideline (SChecklist)." When completing the checklist, please use section and paragraph numbers, rather than page numbers.** Response: As requested, we include the STROBE checklist in the revised manuscript as a Supplement 1. We have referenced section and paragraph numbers as requested.
- **As noted by one reviewer, residual confounding (and/or reverse causation) are possible explanations for the effects seen in the analysis, which limit interpretation of the associations. We'd caution the authors to ensure that the writeup in the paper is not overly conclusive about a causal basis to the associations reported in the analysis - these should be presented as associations/links rather than definitive effects.** Response: We strongly agree that the manuscript should not include causal language as it is largely cross-sectional in nature. We have reinforced this in the Abstract as noted above by including the sentence “These findings must be viewed with caution as residual confounding and/or reverse causation are may contribute to these associations” at line 51 of the revised manuscript. Also, as requested by Reviewer #2, we have specifically addressed the possibility of reverse causality in the Limitations section (Line 465 of revision). This section already indirectly addressed this possibility in stating that we assessed smoking exposure multiple times prior to year 25, but the abdominal measures only once at visit year 25 (see also response to Reviewer #2 below).

**Comments from the reviewers:**

**Reviewer #1:** **I confine my remarks to statistical aspects of this paper. These were well done and I recommend publication.**

**Response:** We greatly appreciate Dr. Flom’s review and are pleased the statistical analyses meet the *PLOS Medicine* standard.

**Reviewer #2:** **Terry and co-authors examined the association between cigarette smoking and intermuscular adipose tissue and attenuation in the well-described CARDIA population. Although the authors used rather unique measures in a large sample, I have a number of concerns specifically focused on the clinical/medical translation of the findings.**

**1)	The nature of the study is observational only, so no causality can be inferred on the basis of this data.**

**Response:** We agree that causality cannot be inferred based on the study design. We have smoking data from across the 25-year span of the CARDIA study, but the CT measures were only performed once, at the end of that period. As recommended by the editors, we stress this point in the Abstract, last sentence of the Methods and Findings section (revision line 51). We also tempered the language used in the Discussion in last two sentences of the first paragraph (revision markup lines 371-374). We further added a direct statement that we cannot rule out reverse causality in the Limitations (line 465).

**2)	The analyses are done cross-sectional meaning that the direction of the effect cannot be explained. Previous studies (e.g., Mendelian Randomization studies) showed that individuals with obesity have a higher propensity to be a current smoker.**

**Response:** We agree with the reviewer that the direction of the association cannot be identified in the present study. As such, we have carefully limited causal language and included statements to that effect as detailed above. We also now cite a recent Mendelian randomization study from UK Biobank with the caveat that the association between smoking and obesity may run in either direction (revision markup lines 375-386). These data support our findings that current smokers and those with higher pack-years exposure, have higher adiposity in the abdomen. Our data suggest that part of this higher abdominal fat is found in muscles as well.

**3)	The clinical relevance of the findings is poorly examined in the present study. For example, there is an approximate 0.4 units difference in IMAT volume between never and current smokers. The impact of this difference on health (e.g., diabetes or CAD risk) is not discussed. A mediation analysis would be helpful to see whether the association between smoking and health outcomes is mediated by the differences observed in the present study. Especially because the authors also showed a lower BMI in the current smokers, this would be interesting. Combining all anthropometric difference in current smokers would provide a clearer image to the impact on diseases like T2D (or Hb1Ac or HOMA-IR, in case they measured this).**

**Response:** We agree that the manuscript findings needed to be put in better context in terms of health risk associated with higher IMAT. We now put the 0.4 cm^3^ difference in IMAT between current smokers and never smokers in context of diabetes prevalence by relating it to our prior, published study (revision line 430-433). We appreciate this suggestion as it bolsters the existing discussion of the risk for diabetes from higher IMAT. We believe that the mediation analyses suggested by the reviewer would be of interest and are appropriate for presentation in a dedicated manuscript.

**4)	It would have been interesting to examine the association of years of smoking cessation and the study outcomes. It seems that past smokers have an IMAT/Attenuation which is a bit in the middle between past en never smokers, but this group is very heterogenous in terms of the duration of smoking cessation. This would give this study a somewhat longitudinal aspect.**

**Response:** We appreciate this thoughtful suggestion and performed the analysis suggested by the reviewer, but, unfortunately, there was no significant association between years since quitting and muscle composition variables. We now report this analysis in Results on line 318 of the revised manuscript markup. As the reviewer surmised, the former smoker group includes a heterogeneous mix of those who smoked remotely and had little exposure in terms of pack-years and those who recently quit or intermittently smoked throughout the study.

**5)	It is unclear to this reviewer what the authors tried to do with the adjustment for the baseline variables (25 years ago!).**

**Response:** The analysis based on smoking habits at CARDIA baseline, 25 years prior, was performed to try to get at the question raised by the reviewer of cart and horse. It has been shown in CARDIA that risk factors measured in participants at baseline (age 18-30) are often the better predictors of health many years later. For instance, in 2007 Loria et al. (JACC 49(20):2013-20) showed that at baseline cigarettes per day (and other risk factors including LDL and glucose) were more strongly associated with prevalent coronary artery calcium (CAC) 15 years later than concurrent measures at the year 15 visit. Although smoking is often a life-long decision that has been made by age 18-30 (the CARDIA baseline age range), we thought it worthwhile looking at those data as well. Moreover, we wanted to test the association of cotinine, as an independent assessment of smoking status, with the abdominal adipose and muscle composition data and the cotinine levels were only measured at baseline.

**Reviewer #3:** **This is an excellent paper on an important topic. I am surprised the analysis has not been done before. The authors have been thorough in their statistical analysis and the discussion is well-written.**

**I only have very tiny amendments:**

**-"type 2 diabetes" rather than "type ii diabetes." (the latter term is older nomenclature) -In the opening sentence of the introduction, change "was" to "is." Alternatively, add the word "historically" to the beginning of the sentence to make the tenses match.**

**Response:** We greatly appreciate the kind words from Reviewer #3! As requested, we have added the word “Historically,” to the beginning of the first sentence in the Introduction to correct the tense issue (line 103 of the revision). We have also changed type II diabetes to diabetes as requested.
