# Supplementary material for: Association of smoking with abdominal adipose deposition and muscle composition in Coronary Artery Risk Development in Young Adults (CARDIA) participants at mid-life: A population-based cohort study
Source: PLoS Med. 2020 Jul 21;17(7):e1003223. doi: 10.1371/journal.pmed.1003223 (PMC7373261; doi:10.1371/journal.pmed.1003223)
Supplement: Attachment — Submitted filename: PLOS Med Editor Response 20200615.docx [file pmed.1003223.s008.docx]

**Email dated June 8, 2020**

**Requests from the editors:**

**Abstract**

- **Could you please provide additional participant demographics?** We now include additional participant information in the Abstract including racial demographics, cities of recruitment, and study history. Lines 37-40 of the revision markup.
- **Please use format 0.xxx for p-values throughout.** As requested, we have revised the p-value formatting in the Abstract, manuscript body and tables.
- **If a prospective analysis plan was used, please include as Supporting Information file.** We now include the CARDIA Study Publications and Presentations document required for approval of CARDIA manuscripts in Supporting Information file 1. We also cite the file (line 153 revision markup) and provide file legends beginning line 506. There were no changes to the analysis plan to disclose.
- **Please provide a space between text and refence brackets.** As requested, we have included the space.
- **Suggest alternative to “svelte figure” and “quitters”.** As suggested, the first sentence of the Introduction has been modified to read “Historically, cigarette advertisements promoted the idea that a lower body weight was a possible beneficial side effect of smoking”.
- **Rephrase “obese, ageing society”.** As requested, the sentence was modified by the deletion of the phrase altogether (line 127 of revision markup).
- **Please provide copies of the questionnaires used in the study or provide citations.** The descriptions of CARDIA clinical data collection are cited as References #31-36. All study forms have been maintained in a consistent format throughout the study. We have included the participant smoking history forms as Supporting Information File 3 as these may be of interest to readers.
- **Please provide exact p-values line 269 and provide data.** As requested, we have included the data and exact p-value now shown on lines 276-279 of the revision markup. Moreover, we have deleted instances of “data not shown” and revised p-value formatting as suggested in text and tables.
- **Please define CRP, CAC and HU in table footnotes.** As requested, we have included these definitions in footnotes of all tables.
- **Please revisit the possibly contentious statement “tobacco industry’s longstanding…”. Suggest deleting the sentence.** As suggested, this sentence has been deleted from the revision along with the reference (line 382 of the revision markup).
- **Please ensure that lines 101-102 reflect content of cited references.** Although these lines do accurately reflect the cited references, we now tone down the sentence to read “Historically, cigarette advertisements promoted the idea that a lower body weight was a possible beneficial side effect of smoking”. We will further revise should the Editors suggest it.
- **At line 471, unless there are surveys documenting the beliefs of “millions of smokers”, please amend the wording.** We have deleted the phrase from the revision (line 482 of revision markup).
- **Please provide full reference #1.** Provided as requested.
- **Please adapt title to non-declarative style and remove word “prospective”.**

As requested, we have modified the title as follows:

Association of smoking with abdominal adipose deposition and muscle composition in Coronary Artery Risk Development in Young Adults (CARDIA) participants at mid-life: a population-based cohort study.

- **Please remove all instances of “[internet]” from reference list.** As requested, we have removed the internet insert.

**Email dated June 15, 2020**

**Requests from the editors:**

**Figures and Graphics:**

1. **Please remove embedded figures and ensure the most up to date versions of figures are uploaded as TIF or EPS files.** We have removed the figures and ensured that the uploaded figures are current. We also retain the figure legends in the revision text at lines 329-338 of clean version of revision.
2. **Please ensure figures are cited in ascending order in main text and refer to figures as Fig 1 etc.** We have revised as requested.
3. **Please ensure that figure legends are placed after the citing paragraph.** We have figures cited as requested.

**Tables:**

1. **Tables 1, 2 and 3 use returns to control text wrapping. Please either split those cells into separate cells or remove the returns.** As requested, I have removed the returns used in the row and column headers for Tables 1, 2 and 3. If any issues remain with these tables, I will gladly reformat further.

**Metadata:**

1. **Please ensure that Data Availability Statement in the submission form identifies how readers can access data.** The data access requirements are stated as requested.

**Article File:**

1. **Please indicate corresponding author in byline with asterisk and email address.** Author so indicated in revision.
2. See #1 above.
3. **The name of co-author James G. Terry does not match across article and submission form. Please match verbatim.** Author is James G. Terry as revised.
4. **Please remove funding sources from manuscript file. Check the financial disclosure field for accuracy.** Funding sources removed from manuscript as requested and accuracy confirmed.
5. **Please remove the competing interests section from the manuscript file.** Done as requested.
6. **Please remove the data availability information from the manuscript file.** Done as requested.
7. **Please ensure your ethics statement is available in the Methods section in its entirety.** Ethics statement is **i**ncluded on line 157-159 of the revision markup.

**References:**

1. **Please ensure the references are in style of PLOS Medicine.** As requested, references are in Journal style.
2. **Please put all references square brackets separated by commas or dashes with no spaces**. References are formatted as requested.
3. **Please make sure references are cited in ascending order in text.** We have reviewed and references are in ascending order.

**Supporting Information:**

1. **Please make sure your supporting information files are uploaded to file inventory and not included in manuscript.** Supporting information files uploaded and named as requested.
2. **Please be sure that supporting information files are named and cited as requested.** Files were named and cited in texted as required.
3. **No generic supporting information references are permitted.** Cited as requested in Methods.
